# Diet and Asthma: Is It Time to Adapt Our Message?

**DOI:** 10.3390/nu9111227

**Published:** 2017-11-08

**Authors:** Laurent Guilleminault, Evan J. Williams, Hayley A. Scott, Bronwyn S. Berthon, Megan Jensen, Lisa G. Wood

**Affiliations:** 1 Priority Research Centre for Healthy Lungs, Hunter Medical Research Institute, University of Newcastle, Callaghan, NSW 2308, Australia; guillel@free.fr (L.G.); evan.j.williams@uon.edu.au (E.J.W.); hayley.scott@newcastle.edu.au (H.A.S.); bronwyn.berthon@newcastle.edu.au (B.S.B.); 2 Priority Research Centre Grow Up Well, Hunter Medical Research Institute, University of Newcastle, Callaghan, NSW 2308, Australia; megan.jensen@newcastle.edu.au

**Keywords:** diet, asthma, inflammation, obesity

## Abstract

Asthma is a chronic respiratory disorder which is associated with airway inflammation. Environmental factors, in association with genetic susceptibility, play a critical role in asthma pathophysiology. Inhaled allergens, smoke exposure, indoor and outdoor air pollution are common triggers of asthma symptoms. Although the role of diet has clearly established mechanisms in diseases such as cardiovascular disease, type 2 diabetes, and cancer, it is not commonly identified as a causal factor in asthma. However, some dietary patterns, such as the Western diet, which includes a high intake of refined grains, processed and red meats, and desserts, have pro-inflammatory effects. On the contrary, the Mediterranean diet, with high intake of fruits and vegetables has anti-inflammatory properties. The influence of food on asthma outcomes is of growing interest, but dietary habits of asthma patients are not commonly investigated in clinical practice. In this review, we focus on the impact of diet on asthma risk and asthma control. We also detail the influence of diet on obese patients with asthma.

## 1. Introduction

Eating is an essential part of life. Beyond the concept of survival, it is well known that food plays an important role in preserving health [1]. The famous Hippocrates’s quote “let food be thy medicine” introduced the idea of the potential impact of food on health. Nowadays, the role of food in chronic diseases, including cardiovascular disease, type 2 diabetes, metabolic syndrome, and cancer, is undisputed [2], and dietary interventions are an important component in managing numerous chronic diseases [3]. The role of diet as a key factor influencing the development of allergic diseases has been described [4]. The direct influence of food on asthma outcomes regardless of allergic status has emerged more recently [5], with dietary factors showing the potential to be directly involved in asthma pathogenesis [6,7]. Obese asthmatics have a distinct phenotype with a unique pathophysiology (low eosinophilic inflammation, low allergen sensitization) [8,9]. The specific role of diet on asthma outcomes in obese patients is also of growing interest due to their limited responsiveness to inhaled corticosteroids, as well as other difficulties faced when managing these patients [10,11]. The key question for clinicians is to know whether changes in the diet could benefit patients with asthma in routine practice. In this review, we will describe the impact of diet on asthma, and identify messages that can be used in asthma management. Given that obese asthmatic patients represent a particular group in terms of inflammation and clinical management, we will also focus on the effect of diet on asthma patients with obesity.

## 2. Dietary Patterns

The literature examining the effect of dietary intake on asthma risk and progression examines both individual nutrients and dietary patterns. Dietary patterns describe the overall habitual intake of food and food groups by individuals and groups, with consideration given to the combination, frequency, quantity, and variety [12]. Dietary pattern analysis provides several advantages in examining the relationship between dietary intake and health outcomes: foods or nutrients are not eaten in isolation, and considerable interactions and synergistic effects exist with common nutrients in foods [13]. Two commonly investigated dietary patterns are the Mediterranean diet (Med-diet) and the Western diet.

**Figure 1:**
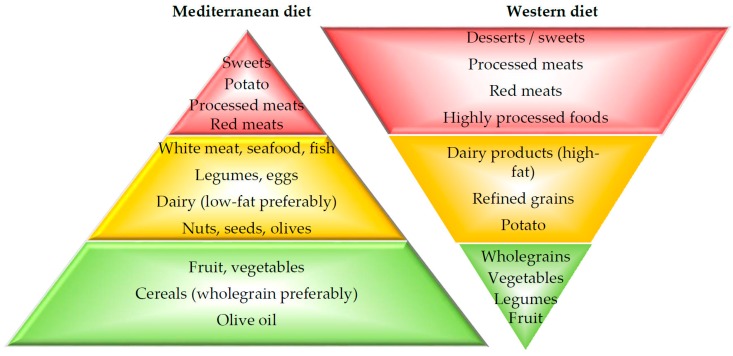
Characteristics of Mediterranean and Western diets. The Mediterranean dietary pattern is based on a variety of fruits, vegetables and wholegrain cereals. In contrast, the Western diet is dominated by convenience and highly processed foods, and includes a high intake of refined grains, processed and red meats, desserts and sweets, fried foods, and high-fat dairy products, with a low intake of fruits and vegetables.

The Mediterranean dietary pattern was identified in the 1950’s and 1960’s along the coastal regions of southern European countries, including Italy and Greece, and was associated with a lower mortality rate from coronary heart disease [14]. Current recommendations for the Med-diet promote meals based on a variety of fruits, vegetables and wholegrain cereals [15]. In contrast, the Western diet, prevalent in developed countries, is dominated by convenience and highly processed foods, resulting in high intakes of refined grains, processed and red meats, desserts and sweets, fried foods, and high-fat dairy products, with low intake of fruits and vegetables (Figure 1) [12].

One of the key differences between the Med-diet and Western diet, is the amount of fruits and vegetables that are consumed. Increased intakes of fruit and vegetables are recommended for the general population due to their abundance of vitamins, minerals, fibre and non-nutritive phytochemicals such as antioxidants, polyphenols, flavonoids and carotenoids, and low energy content [16]. Fruits and vegetables feature prominently in the dietary guidelines for many countries, including Australia [17], Canada [18], USA [19], United Kingdom [20], France, Germany and Spain [21], with recommendations ranging from 5–7 serves per day. However, the intake in many Westernised countries is inadequate with mean national intakes meeting recommended targets in only 0.4% of the global population for fruits and vegetables [22].

Another key difference between the Med-diet and Western diet, is the amount of seafood consumed. Seafood is the primary food source of long-chain omega-3 (LC *n*-3) polyunsaturated fatty acids (PUFA), docosahexaenoic acid (DHA), eicosapentaenoic acid (EPA), and docosapentaenoic acid (DPA), with fatty fish such as salmon, mackerel, and sardines providing higher levels (1.7–2.2 g/100 g) [23]. Western populations are estimated to have a low intake of LC *n*-3 [23] with few reaching the targets for chronic disease reduction of 0.6 g/day for men and 0.4 g/day for women [17], which can be achieved by consuming the recommended two or more serves of (preferably oily) fish per week [24]. Fish oil supplements contain approximately 30–60% EPA/DHA, and are often used to achieve higher LC *n*-3 intakes [23].

In general, the Med-diet is believed to have health benefits, largely attributed to the content of fibre, antioxidants, protein, and moderate amounts of fat-predominantly from mono-unsaturated (MUFA) and omega-3 PUFA. On the other hand, consumption of a Western diet is believed to have detrimental consequences, due to the excess consumption of total energy, saturated fat and omega-6 PUFA, sugar and sodium, combined with the low intakes of omega-3 PUFAs, protein, fibre, micronutrients (including folate and magnesium), antioxidants, and phytochemicals (e.g., carotenoids, flavonoids) [25].

## 3. Diet and Inflammation

### 3.1. Diet and Systemic Inflammation

Systemic inflammation can be a feature of asthma, particularly in the neutrophilic inflammatory phenotype [26,27], and is associated with worse disease outcomes, such as poorer lung function [28,29,30], increased frequency of exacerbations [31,32] and increased airway inflammation [26,31].

**Figure 2:**
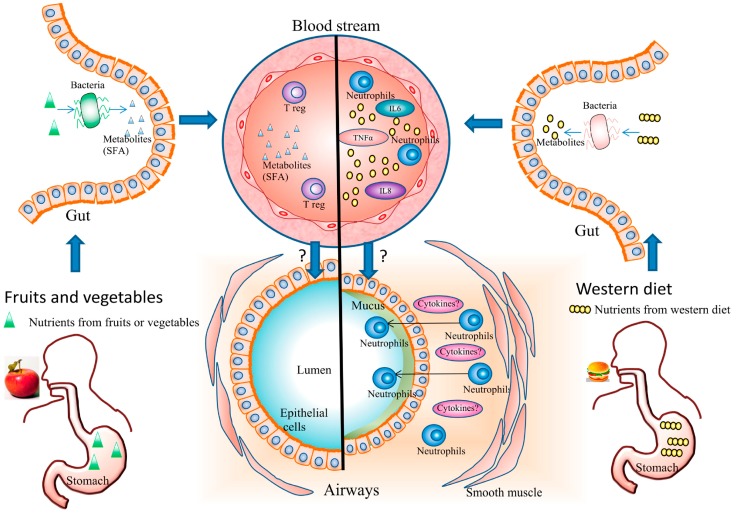
Systemic and airway effects of dietary patterns on asthma. The Western diet promotes a pro-inflammatory environment and causes an increase in airway inflammation. Fruit and vegetable consumption has systemic anti-inflammatory properties, with a decrease of pro-inflammatory cytokines in plasma. Fruit and vegetables are also associated with lower airway inflammation and a reduction of neutrophils in asthmatics. Gut microbiota plays a role in immune response to diet in asthma. Metabolites such as short-chain fatty acids (SCFA) (including ω-3 fatty acids) that have immunomodulatory effects are produced in high amounts after fruit and vegetable intake. The western diet altered microbiota composition and potentiate inflammation.

Dietary intake has been shown to modify systemic inflammation (Figure 2). The Western diet has been thought to promote a pro-inflammatory environment due to factors such as lack of antioxidants, which increases susceptibility to oxidative stress, and abundance of saturated fatty acids, which can lead to innate immune activation, via activation of receptors such as toll like receptor 4 (TLR4), which stimulates the NF-κB inflammatory cascade [33,34,35]. In contrast, the Med-diet is thought to promote an anti-inflammatory environment [36,37], due to the presence of anti-inflammatory nutrients such as unsaturated fatty acids e.g., MUFA, omega-3 PUFA and antioxidants [36,37]. These anti-inflammatory effects have been shown in a recent meta-analysis noting significant decreases in plasma C-reactive protein (CRP), IL-6 and intracellular adhesion molecule-1 (ICAM-1) in healthy individuals following a Med-diet [38].

### 3.2. Diet and Airway Inflammation

Chronic inflammation of the airways is a key component of asthma [39], which may be modulated by dietary intake [40,41,42,43,44]. High fat intake, a characteristic of the Western diet, can cause an increase in airway inflammation. Consumption of a high-fat mixed meal has been shown to increase sputum neutrophils 4 h post-meal in patients with asthma [40], as well as activation of a number of genes in sputum involved in “immune system processes”, such as TLR4, indicating an increase in airway inflammation [44]. Reduction of dietary saturated fat intake was associated with a reduction in neutrophilic airway inflammation in asthmatics [45]. In adults with severe asthma, higher fat and lower fibre intakes have been associated with increased eosinophilic airway inflammation [42].

In contrast, fruits, vegetables and their antioxidants may lower airway inflammation [41,43]. Fruit and vegetable intake was inversely associated with IL-8 protein in nasal lavage of asthmatic children [43]. In asthmatic adults, intake of tomato juice, which is abundant in the antioxidant lycopene, reduced airway neutrophil influx and sputum neutrophil elastase activity after just seven days of supplementation [41]. The link between systemic inflammation and airway inflammation induced by diet is still unknown.

### 3.3. Diet and Gut Microbiota

The gut microbiome is responsible for the production of some of the most important metabolites that impact on immune and metabolic responses and can be modulated by different dietary patterns [46]. Fruit and vegetables are a rich source of dietary fibre, which is fermented by gut bacteria, to produce metabolites such as short-chain fatty acids (SCFA). These metabolites have immunomodulatory mechanisms, such as activation of metabolite-sensing G-protein-coupled receptors (GPCRs), and epigenetic and gene transcription modulation and have been shown to affect airway inflammation and airway reactivity in animal models of allergic airways disease [47,48,49]. High fibre diets have been shown to protect against airway allergic responses, associated with increased colonic Bacteroidetes and Actinobacteria species, and decreased Firmicutes and Proteobacteria [50,51]. In mice, a high dietary fibre intake during pregnancy protects against the development of airway disease in the offspring, with marked effects on the composition of the gut microbiota [52].

A high-fat diet also alters gut microbiota composition, particularly the expansion and colonization of invasive bacteria, reduction in protective bacteria, and reduction in SCFA concentrations, supporting a potential role in inflammation and immune response [53,54,55]. The role of high fat diets on gut microbiota and asthmatic immune responses is unknown.

## 4. Diet and Risk of Asthma

The influence of dietary intake on asthma risk has been examined in many studies over the last two decades. Mediterranean diets and Western diets, whole foods and individual nutrients, have been found to have an influence on asthma risk, at various stages of the life cycle (Table 1).

### 4.1. Maternal Diet during Pregnancy

Asthma susceptibility is likely determined early in life for a large majority of patients [138]. In-utero exposures including tobacco smoke, maternal obesity, prenatal infection and exposure to microbial products can influence childhood respiratory disease risk [139,140]. The impact of maternal diet during pregnancy on the foetus and future child health is a recurrent question [141,142].

#### 4.1.1. Dietary Patterns: No Evident Effect

Cross-sectional studies have shown a protective effect of the Med-diet during pregnancy on infant wheeze in the first year of life and asthma development in childhood [58,59,60]. However the data from cohort studies are inconsistent. In one study, a high quality Med-diet consumed during pregnancy was found to be protective against persistent wheeze at 6.5 years of age [61]. However, in two recent prospective cohort studies, adherence to the Med-diet was not associated with a reduced risk of wheeze in the first year of life, at 1.5 years or four years of age [62,63]. The results of a Med-diet intervention in a sample population of pregnant women in the UK are expected soon [143].

Fruit and vegetable consumption during pregnancy does not appear to have an impact on childhood asthma. As with the Med-diet, cross-sectional studies suggest a beneficial effect of fruit and vegetable intake during pregnancy on wheeze and/or asthma in childhood [58,72,73]. However, a meta-analysis that pooled the available cohort studies found that maternal consumption of fruits and vegetables during pregnancy was not associated with risk of wheeze or asthma in the offspring [61,63,74,75,76,77]. In cross sectional studies, fast food consumption at least three times a week during pregnancy increased the odds of childhood wheeze by 2.17–3.96, compared to a consumption less than once a week [60,86,87]. In contrast, cohort studies found no association between the Western diet or a high intake of fast food consumption during pregnancy and childhood wheeze [63,91,92,93]. In a meta-analysis, no association was found between meat intake during pregnancy and childhood wheeze and asthma, but one cohort study found that high intakes of ‘processed’ meat during pregnancy were associated with an increased risk of wheeze in the first year of life with an odd ratio (OR) of 1.18 (95% confidence interval (CI) 1.02–1.37) [62,101].

Given the inconclusive data, no message can be clearly delivered to pregnant women regarding dietary patterns or nutrients other than the current dietary guidelines.

#### 4.1.2. Vitamin D and E and Fish Oil: Beneficial

Individually, intake of vitamins A (and carotenoids), B, or C during pregnancy are not associated with wheeze and/or asthma in childhood [75,114,115,116,119,120,121,122]. However, vitamin D intake during pregnancy was associated with a reduction in childhood wheeze (OR ranged from 0.58, 95% CI 0.38–0.88 to 0.81; 95% CI, 0.67–0.98) in meta-analyses [101,123]. A similar effect has been observed with vitamin E (OR 0.54, 95% CI 0.41–0.71) [75,101,121,122,127]. However, no effect of vitamin D or E on risk of childhood asthma has been found [75,101,115,124,125,127]. Although no guidelines have been published on the necessity of vitamin supplementation in pregnant women for decreasing asthma risk, general guidelines used for vitamin D supplementation during pregnancy should be applied in all pregnant women by respiratory medicine physicians [144].

No link has been observed between fish intake during pregnancy and wheeze and/or asthma in childhood in most studies, nor in a meta-analysis [74,77,92,102,103,104,105,106,107,108]. However, fish oil supplementation during pregnancy has been shown to decrease wheeze or asthma in childhood [130,131,132]. In an RCT, fish oil supplementation (2.7 g/day) during pregnancy, containing LC *n*-3 PUFA, reduced the risk of asthma development in the offspring by 63% (95% CI: 8–85%) and this protection appears to be maintained into adulthood [130,131]. Latest evidence from an RCT also found a reduction in asthma or wheeze in children from mothers using fish oil supplements (2.4 g/day) during pregnancy [132].

### 4.2. Children

#### 4.2.1. Breastfeeding: Beneficial

The World Health Organization and the American Academy of Paediatrics emphasize the value of breastfeeding for infant health. The strong protective effect of breastfeeding on asthma development is clearly established. This effect is particularly observed at age 0–2, and decreases over time but is maintained after seven years of age [56]. Compared to no breastfeeding, any duration of breastfeeding was inversely associated with asthma in children aged 0–2 years, 3–6 years, and older than 7 years, with an OR of 0.65 (95% CI 0.51–0.82), 0.79 (95% CI 0.68–0.91) and 0.86 (95% CI 0.77–0.96), respectively [56].

#### 4.2.2. Fruit and Vegetables: Beneficial

Fruit and vegetable intake in childhood appears to have a beneficial effect on wheeze and asthma risk. In a meta-analysis, fruit intake had an inverse association with wheeze and asthma with a relative risk (RR) of 0.81 (95% CI: 0.74–0.88) and 0.90 (95% CI: 0.86–0.94) respectively [78]. Vegetable intake was also inversely associated with wheeze and asthma with a RR of 0.88 (95% CI: 0.79–0.97) and 0.91 (95% CI: 0.82–1.00), respectively [78]. Our group reported a beneficial effect of fruits or vegetables on asthma [80]. In the only cohort study included in this meta-analysis, fresh fruit consumption at early age and long-term fruit intake, but not vegetable intake, was associated with reduced asthma symptoms (OR = 0.93, 95% CI 0.85–1.00 and 0.90, 95% CI: 0.82–0.99) [79]. Another cohort study that was subsequently published confirmed the protective effect impact of fruit and vegetable consumption on asthma [81]. In order to decrease the risk of childhood asthma, fruit and vegetable intake should be encouraged particularly for the youngest children.

#### 4.2.3. Dietary Patterns: Med-Diet–No Effect, Western Diet–Detrimental

A protective effect of the Med-diet has been observed only in cross-sectional studies [59,64,65,66,67,68]. A prospective study did not find an association between the Med-diet and current wheezing at 1.5 and four years of age [63]. The majority of data from prospective and cohort studies describe a protective effect of fish consumption on asthma or wheeze [95,105,109,110,111,112]. Of two meta-analyses assessing the effect of fish consumption on asthma, one found a protective effect of childhood intake of fish [108,113].

In Western society, children have high intakes of ‘fast food’, and this increases further in adolescence. However, fast food and western diets should be limited because of their negative impact on asthma risk [88]. Consumption of fast food and meat at least three times a week is significantly associated with wheeze at four years of age [63]. In addition, fast food consumption in childhood may decrease the protective effect of breastfeeding on asthma risk [89]. More generally, a Western diet that is high in fat and processed foods has been shown to increase the risk of asthma or wheeze in childhood [94,95]. Recently, two studies have found an association between sugar-containing beverages (fruit drinks and soda) and asthma [96,97].

#### 4.2.4. Vitamins and Fish Oil: Inconclusive Data

In early childhood, two RCTs have shown that fish oil intake had no effect on asthma risk [133,134], while one RCT found a protective effect of fish oil on wheeze [134]. In the most recent meta-analysis, PUFA consumption was not found to have any beneficial effect on asthma [135].

Although antioxidant and vitamin deficiency has been found in asthmatic children, β-carotene (vitamin A) or vitamin D supplementation does not appear to reduce the risk of asthma or wheeze [117,118,126]. Vitamin C consumption has been described as protective for childhood wheeze, but the results were extrapolated from fruit rich in vitamin C [145,146].

### 4.3. Adults

Understanding the link between dietary intake and asthma risk in adults is difficult, due to the multitude of clinical and inflammatory phenotypes that the disease encompasses and considering disease modifying effects of diet in terms of prevention versus management of established disease.

#### 4.3.1. Breastfeeding: No Effect

The effect of breastfeeding in childhood does not appear to be sustained in adulthood. In one cohort study with a follow-up from birth to 20 years, breastfeeding was not associated with a reduction in adult asthma [57].

#### 4.3.2. Fruits and Vegetables: Beneficial

Although the level of evidence is low, fruit and vegetable consumption seems to decrease asthma risk. Indeed, fruit is inversely associated with asthma in cohort studies and the strongest association has been observed with intake of apples and oranges [69,82,83]. The positive effect of vegetables on asthma has indirectly been suggested in a cohort study analysing the effect of flavonoids [83], and also in several cross-sectional studies [84,85], while other studies have found no association [70]. To our knowledge, no studies have specifically evaluated the effect of the Med-diet on asthma development in adults but one cross-sectional and two cohort studies found no association between fish and fruit and/or vegetable intake and adult asthma [69,70,71]. A recent review did not find evidence to support an association between a Western diet and incident adult asthma [90]. However, sugar-sweetened beverage intake at least two times per day was associated with adult asthma in three cross-sectional studies with an OR ranging from 1.26 (95% CI: 1.01–1.58) to 1.66 (95% CI: 1.39–1.99) [98,99,100]. The dichotomy between Western diet and Med-diet does not reflect the complexity of dietary intake. The dietary may be radically different from one day to another. A French study analysed the composite score Alternate Healthy Eating Index 2010 (AHEI-2010) that measures the diet quality in asthma control [147]. The investigators observed an asthma control improvement with an improvement in AHEI-2010.

#### 4.3.3. Vitamins and Fish Oil Supplementation: Inconclusive Data

Although vitamin A, C and E deficiency has been suggested to increase the risk of asthma in adults [148,149,150], the effects of supplementation are not conclusive due to a low evidence level. One case control and one prospective study found a potential beneficial effect of vitamin E from dietary sources, while vitamin E supplementation was not protective [128,129].

A meta-analysis, published in 2013, found no association between fish or fish LC *n*-3 PUFAs and the risk of asthma [113]. However, the analysis was based on only two studies: a case control study and a prospective study [136,137]. Although the case-control study (525 subjects) observed no association between LC *n*-3 PUFAs and asthma risk [136], the prospective study (4162 subjects with 446 incident cases of asthma) found that the highest quintile of LC *n*-3 PUFAs intake was inversely associated with the risk of asthma (HR 0.46, 95% CI: 0.33–0.64) [137]. Other studies are warranted to form definitive advice in this area.

## 5. Diet and Asthma Control

According to the Global Initiative for Asthma (GINA), asthma control has two domains: symptom control and future risk of adverse outcomes (such as exacerbations and lung function decline) [151]. Beyond traditional asthma pharmacotherapy usually used in asthma patients, numerous non-pharmacological treatments (such as physical activity, avoidance of allergens, smoking cessation) are recommended to enhance asthma control. In the GINA report, a healthy diet with fruits and vegetables is encouraged in asthma patients for its general health benefits [151]. However, in addition to its general health benefits, evidence suggests that diet could play a role in asthma control (Table 2).

### 5.1. Pregnancy: Studies Are Needed

The scientific evidence for a benefit of dietary interventions on asthma control in pregnancy is limited. In a cross-sectional study of 180 asthmatic pregnant women, those with uncontrolled asthma had higher energy-adjusted intakes of saturated fat, total carbohydrate, and sugar compared with women with controlled asthma; notably, they also had higher intakes of MUFA and fibre [152]. Further studies on the impact of diet on asthma in pregnant women are warranted.

### 5.2. Children

#### 5.2.1. Med-Diet, Fruit and Vegetables: Beneficial

In a prospective study performed in children 1–5 years of age, a nutritional education programme based on the Med-diet recommendations led to a significant reduction in asthma exacerbations after 1 year compared to the previous year and decreased use of asthma medications (inhaled corticosteroids and short acting bronchodilators) [153]. Another study found that adherence to the Med-diet was inversely associated with asthma symptoms [154]. The direct effect of fruit and vegetable intake on asthma control in children has not been adequately studied. Providing free fruit at school did not have a significant impact on asthma control, however this is not surprising as fresh fruit intake increased marginally from 1.3 to two servings per day [155]. In a multicentre cross-sectional study, fruit intake at least three times a week was associated with a reduction in current wheeze and severe symptoms of asthma (OR 0.87 (95% CI 0.80–0.95) and 0.86 (95% CI 0.76–0.97), respectively), in children aged 6–7 years [156]. A similar association was observed for vegetable intake. The positive impact of fruits and vegetables was also observed in adolescents. RCTs are warranted to elucidate the direct effects of the Med-diet and fruit and vegetable intake on asthma control. A combination of fruit plus vegetable concentrate, fish oil and probiotics has demonstrated promising results in asthmatic children aged 10–12 years with an improvement in pulmonary function parameters and reduction in the use of short-acting inhaled bronchodilators and inhaled corticosteroids [157].

Key features of the Western diet have been associated with negative asthma outcomes. Salty-snack (foods high in salt and fat and low in vitamins and antioxidants) and fast food consumption ≥3 times a week has been associated with the presence of asthma symptoms, even after adjustment for sex and body mass index (BMI) [156,158,159].

#### 5.2.2. PUFA: Conflicting Data

Findings from studies describing the role of fish oil LC *n*-3 PUFA on asthma control in children are inconsistent. A significant reduction in wheeze, bronchodilator use and nocturnal coughing has been observed in children aged 18 months, who have the highest blood concentrations of omega-3 PUFAs [160]. However, in a 2002 meta-analysis, fish oil supplementation did not have any effect on asthma control [161]. More recently, a significant 10% reduction in the prevalence of cough was found in atopic children, younger than three years, who were given omega-3 fatty acid supplements compared to children who were given placebo [162]. Another trial using daily consumption of a nutritional formula enriched in EPA (3 g/day), gamma-linolenic (GL) acid (1.6 g/day) and antioxidants showed improvement in asthma symptom-free days, but there was no difference compared with placebo [163].

#### 5.2.3. Vitamin C and D: Possible Benefit

The role of vitamins on asthma control is still debated in children. Although folate deficiency appears to be associated with severe exacerbations, no clinical study has assessed the efficacy of folate supplementation in improving asthma control [164,165]. Vitamin C supplementation (0.2 mg/day) has been shown to provide small improvements in asthma control in a cross-over trial, hence a definitive RCT is warranted to confirm this result [166]. In an RCT, monthly doses of 60,000 intravenous units (IU) vitamin D significantly reduced the number of exacerbations, requirement for steroids and emergency visits in school-aged children compared to placebo [167]. In this study, asthma control was achieved earlier in subjects who received monthly vitamin D compared, with those who received placebo. However, vitamin D supplementation does not seem to have a significant effect on subjective asthma control and oral steroid intake in preschool-aged children; however, larger adequately powered trials are required [168,169].

### 5.3. Adults

#### 5.3.1. Fruit and Vegetables: Beneficial

Although a case-control study suggested that high adherence to the traditional Med-diet increased the likelihood of well controlled asthma, a small RCT of 38 patients concluded that multiple consultation sessions with a nutritionist had no impact on asthma control despite an increase in the Med-diet score [170,171]. On the other hand, high intakes of raw vegetables (>5 units/week) and fruit (particularly citrus fruit) has been associated with well controlled asthma [172,173]. In an RCT, high fruit and vegetable intake (≥5 servings vegetable and two servings fruit daily for 14 weeks) was associated with fewer exacerbations and improved asthma control compared with low fruit and vegetable intake (≤5 servings vegetable and two servings fruit daily) [174]. Therefore current evidence suggests that fruit and vegetable intake can have a significant effect on asthma control in adults, thus increased consumption should be encouraged in patients with asthma.

#### 5.3.2. Vitamins or Fish Oil Supplementation: No Effect

Conversely, there is a paucity of evidence supporting an effect of individual antioxidants on asthma control. Although folate deficiency appears to be related to asthma control, being associated with a significant increase in episodes of shortness of breath in adult asthmatics [175], the effect of folate supplements is still unknown. RCTs have failed to demonstrate an effect of vitamin C, D, or E supplements on asthma control [176,177,178], despite beneficial effects on other asthma outcomes. In an open-label study, a combination of vitamins A, B6, C and E, fish oil, calcium, zinc, and selenium increased asthma control and health-related quality of life at the end of the study [7].

Fish oil alone does not seem to have a significant impact on asthma control in adult asthmatics [161]. However, EFF1009, a medical food emulsion containing GL acid and EPA, leads to improved quality of life and asthma control and decreased reliance on rescue medication [179,180].

#### 5.3.3. Western Diet: Detrimental

A number of studies demonstrate that a Western diet has a negative impact on asthma control [181,182,183]. A high intake of pizza/salty pies, dessert and cured meats has been associated with an increased risk of frequent asthma attacks (OR 1.79, 95% CI 1.11–3.73) [181]. In a French prospective study, cured meat intake was associated with worsening asthma symptoms [182]. A link between fast food consumption and breathlessness has also been observed [183].

## 6. Diet and Lung Function in Asthma Patients

### 6.1. Children

#### 6.1.1. The Impact of Breast Feeding on Lung Function Is Low

As demonstrated in a recent systematic review, breastfeeding has a protective effect on forced expiratory volume in 1 s (FEV_1_) and forced vital capacity (FVC) in healthy children [184]. However, the evidence of a potential effect of breastfeeding on lung function in asthmatic children is still low. No association has been observed between breastfeeding and bronchial hyperresponsiveness (BHR) in children [185,186]. A study found an increase in the FEV_1_/FVC ratio in breastfed children with asthma compared to non-breastfed children with asthma [187]. This lack of association may be due to technical issues, as the effect of breastfeeding on lung function in children aged less than four years is hard to assess and after this age, the effect of breastfeeding may diminish or disappear.

#### 6.1.2. Med-Diet and Lung Function: Beneficial Effect

Research regarding the impact of the Med-diet on lung function in children is conflicting [43,188]. Although a cross-sectional study found no effect of the Med-diet on FEV_1_, lung function parameters (FVC, FEV_1_) were improved with the Med-diet [43,188]. No specific effect on lung function has been found for fruit or vegetable intake in children, although the level of evidence is low (Table 3) [43]. Interestingly, in a RCT performed in asthmatic children, the improvement in FEV_1_ was significantly better in those who were given a supplement containing fruit and vegetable concentrate, fish oil and probiotics than those who received a placebo (107 mL vs. 40 mL) [157]. However, the specific effect of each component contained within the combination remains unknown.

#### 6.1.3. Vitamin C and E and Lung Function: A Low Beneficial Effect

Vitamin A supplementation has not shown an association with lung function in asthma patients [118]. In asthmatic children the effect of vitamin C supplementation, on FEV_1_ may be age dependent [166]. However, the methodology of the RCTs was poor and insufficient to draw a conclusion [189]. Although vitamin D deficiency is correlated with pulmonary function deficit in childhood asthma, intervention with vitamin D supplementation does not seem to have a beneficial impact on lung function [190,191,192]. In a meta-analysis published in 2014, no evidence for a positive effect of vitamin E on lung function was observed. This meta-analysis did not include an RCT, published the same year, that found that FEV_1_ and FEV_1_/FVC improved significantly after supplementation with vitamin E (50 mg/day) but not after placebo in children with mild to moderate asthma [193]. In a meta-analysis, *n*-3 LCPUFA supplementation does not have an impact on lung function, except for exercise-induced asthma, but the studies included had small sample sizes [194].

### 6.2. Adults

#### 6.2.1. Fruit and Vegetable Intake: Beneficial

The Med-diet has no effect on lung function in asthmatic adults [43,170,171,188]. In a RCT performed on adults with asthma, a high fruit and vegetable diet for 14 days led to an improvement in FEV_1_ and FVC, whereas no change was observed in patients following a low fruit and vegetable diet [174]. A cross-sectional study reported that adults with severe asthma had lower fibre intakes than healthy controls, which were related to lower lung function [42]. In asthmatic adults, a beneficial effect on lung function has been suggested for soy genistein in a cross-sectional study [195], while a soy isoflavone supplement had no effect on lung function in a RCT [196].

#### 6.2.2. Vitamins and Lung Function: A Low Positive Impact with Vitamin C

Vitamin B supplementation has not shown an association with lung function in asthma patients [175]. A meta-analysis of observational studies found a very small effect of vitamin C supplementation on FEV_1_ in adult asthmatics [197]. Similarly to children, vitamin D supplementation does not seem to have a beneficial impact on lung function in adults [190,191,198].

#### 6.2.3. Meat and Lung Function: No Impact

In a European study, a diet high in meat and potatoes had no impact on lung function in adult asthmatics [71]. However, a single high-fat meal has been shown to negatively influence post-bronchodilator FEV_1_ in adults with asthma [40].

## 7. Diet and Obese-Asthma Phenotype

Primary obesity is attributable to a chronic energy surplus, through excessive caloric intake and reduced energy expenditure. In Western societies, the obesogenic environment is awash with energy-dense nutrient-poor foods, with obesity affecting one in four adults in the UK, Australia, and Canada, and one in five in France and Germany [199].

Obesity prevalence is even greater in the asthmatic population, affecting up to 58% of adults and 28% of children [200]. Obesity has been associated with poorer asthma outcomes, including worse asthma control, increased exacerbations, increased use of medication, reduced lung volumes, and poorer quality of life [201,202]. Consuming an energy-dense, high fat, and low-fibre diet and non-adherence to dietary guidelines is associated with a significantly higher risk of obesity [203].

The few weight loss RCTs that have been conducted to date in obese children and adults with asthma have demonstrated marginal improvements in asthma control, quality of life and lung function [204,205,206,207]. Two small trials of nutritionist-led dietary programs achieved clinically significant weight loss in obese children, observing improvements in quality of life, asthma control, and systemic inflammation, and fewer events requiring rescue short acting beta agonists (SABA) compared to the control group over 10 and 28 weeks [206,207].

A small three-arm trial randomised overweight/obese adults with asthma to either dietary intervention (low-calorie diet, with two meal replacements per day), exercise intervention (group personal training session once per week, plus gymnasium membership), or a combination of both [45]. The diet ± exercise groups achieved significant weight loss over 10 weeks vs. exercise alone. Improvements in lung volumes and quality of life were observed for all groups, whilst asthma control and systemic inflammation improved in the diet and combined groups only [45]. An earlier RCT utilised an eight week very-low-calorie-diet as part of a 14 week intervention, which induced weight loss of 14.5% (vs. 0.3% in control group) and demonstrated significant improvements in lung function, dyspnoea, and rescue medication use in the intervention vs. control group [208].

Although the small trials available to date provide promising results, larger clinical trials with longer follow-up are needed to investigate the ideal dietary intervention to improve asthma outcomes in obese adults and children with asthma.

## 8. How to Change the Patients’ Behaviour?

Given the potential impact of healthy food on general health and asthma outcomes in asthmatic patients, a message about diet should be delivered to the patient. However is this enough to change the patients’ behaviour regarding diet? As patient education is used to improve therapeutic adherence, a structured intervention is needed to change patients’ diets and physical activity levels. Behavioural therapy is likely to be a necessary component of such interventions, as lifestyle changes are difficult to adopt and maintain. The dietary approach to stop hypertension (DASH) is a dietary pattern characterised by an emphasis on healthy food, with key dietary goals including: consumption of 7–12 servings of fruit and vegetables, as well as two to four servings of low-fat/fat-free dairy products, total fat grams at 27% of estimated energy needs and ≤2300 mg of sodium per day [209]. Adoption of the DASH diet in a 6-month intervention (3-month intensive stage and 3-month maintenance stage with support by behavioural coaches who were dietitians) was feasible and acceptable to adults with uncontrolled asthma [210] and led to small improvements in diet quality and asthma control [211]. In children, to durably change dietary behaviours, the child or adolescent should be actively involved. The I Can Control Asthma and Nutrition Now (ICAN) program, based on nutrition and weight management education, as well as asthma education, has demonstrated promising preliminary results in improving nutrition and asthma health outcomes in high school students [212]. School-based nutrition programs should be assessed in asthmatic children [213]. Incorporation of a multi-disciplinary team including dietitians and exercise physiologists into asthma clinics and services may be a positive strategy to facilitate the adoption and maintenance of beneficial eating and exercise habits in asthma patients.

## 9. Conclusions

In conclusion, higher intakes of fruits and vegetables may have a positive impact on asthma risk and asthma control. In a recent overview, the European Academy of Allergy and Clinical Immunology (EAACI) concluded that the literature supports recommendation in clinical practice to increase the net intake of fruits and vegetables as a way of reducing the risk of asthma, particularly in children [214]. The effect on asthma control is also promising, but well-designed RCTs are warranted to formulate official guidelines. Western diets likely have a negative impact on asthma but the level of evidence is still low. The synergistic effect of a healthy diet and exercise could be an effective way to improve asthma outcomes, and the combination of these two non-pharmacological interventions needs to be thoroughly evaluated. Dietary intervention, based on evidence-based guidelines, should be incorporated into the routine clinical management of patients with asthma, in order to achieve overall health benefits and disease management.

## Figures and Tables

**Table 1:** Diet and risk of asthma or wheezing. 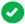 Beneficial effect; 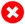 negative effect; 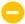 No effect; ? = no data. Very strong evidence is defined as data obtained in meta-analysis of randomized controlled trials (RCTs); Strong evidence is defined as data obtained in individual RCT; Low evidence is defined as data obtained in individual prospective studies or meta-analysis of prospective studies; Very low evidence is defined as data obtained in individual cross-sectional or case-control studies, or meta-analysis of cross-sectional or case-control studies. In case of conflicting results between studies, data from the studies with the most robust methodology are used to define the effect of diet on asthma risk. * One Meta-analysis found a negative association with asthma or wheeze and one found no association; ** conflicting results in cross-sectional studies; *** from diet but not from supplementation.

Diet	Diet During Life Stages					
	Pregnancy		Childhood		Adulthood	
	Effect	Evidence	Effect	Evidence	Effect	Evidence
Post-natal breast feeding			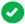 [56]	Very strong	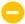 [57]	Low
Mediterranean diet	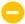 [58,59,60,61,62,63]	Low	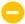 [59,63,64,65,66,67,68]	Low	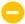 [69,70,71]	Low
Fruit	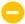 [58,61,63,72,73,74,75,76,77]	Low	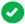 [78,79,80,81]	Low	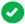 [69,82,83]	Low
Vegetables	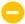 [58,61,63,72,73,74,75,76,77]	Low	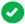 [78,79,80,81]	Low	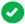 ** [70,83,84,85]	Very low
Fast food	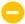 [60,63,86,87]	Low	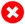 [63,88,89]	Low	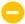 [90]	Low
“Western” diet	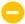 [91,92,93]	Low	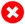 [88,94,95,96,97]	Very low	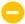 [90,98,99,100]	Low
Meat	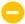 [62,101]	Low	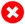 [63]	Low	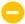 [90]	Low
Fish	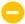 [74,77,92,102,103,104,105,106,107,108]	Low	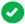 * [95,105,108,109,110,111,112,113]	Low	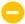 [69,70,71]	Low
Vitamin A	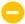 [114,115,116]	Low	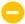 [117,118]	Low	?	?
Vitamin B	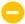 [114,119,120]	Low	?	?	?	?
Vitamin C	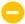 [75,114,115,121,122]	Low	?	?	?	?
Vitamin D	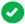 [101,123,124,125]	Very Strong	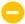 [126]	Low	?	?
Vitamin E	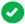 [75,101,115,121,122,127]	Low	?	?	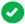 *** [128,129]	Low
LC *n*-3 PUFA (Fish oil)	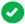 [130,131,132]	Strong	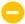 [133,134,135]	Very Strong	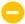 [113,136,137]	Low

**Table 2:** Diet and asthma control. 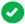 Beneficial effect; 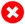 negative effect; 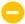 No effect; ? = no data. Very strong evidence is defined as data obtained in meta-analysis of RCTs; Strong evidence is defined as data obtained in individual RCT; Low evidence is defined as data obtained in individual prospective studies or meta-analysis of prospective studies; Very low evidence is defined as data obtained in individual cross-sectional or case-control studies, or meta-analysis of cross-sectional or case-control studies. In case of conflicting results between studies, data from the studies with the most robust methodology are used to define the effect of diet on asthma control.

Diet	Childhood		Adulthood	
	Effect	Evidence	Effect	Evidence
Mediterranean diet	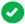 [153,154]	Low	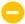 [170,171]	Strong
Fruit	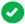 [155,156]	Very low	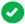 [174]	Strong
Vegetables	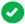 [156]	Very low	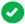 [174]	Strong
Fast food	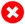 [156,158,159]	Very low	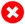 [183]	Very low
“Western” diet	?	?	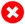 [181]	Very low
Meat	?	?	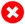 [182]	Low
Fish	?	?	?	?
Vitamin A	?	?	?	?
Vitamin B	?	?	?	?
Vitamin C	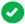 [166]	Low	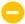 [176]	Strong
Vitamin D	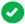 [167]	Strong	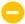 [178]	Strong
Vitamin E	?	?	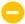 [177]	Strong
LC *n*-3 PUFA (Fish oil)	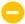 [160,161,162,163]	Strong	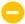 [161]	Very strong

**Table 3:** Diet and lung function in asthma patients. 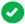 Beneficial effect; 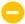 No effect; ? = no data. Very strong evidence is defined as data obtained in meta-analysis of RCTs, Strong evidence is defined as data obtained in individual RCT; Low evidence is defined as data obtained in individual prospective studies or meta-analysis of prospective studies; Very low evidence is defined as data obtained in individual cross-sectional or case-control studies, or meta-analysis of cross-sectional or case-control studies. In case of conflicting results between studies, data from the studies with the most robust methodology are used to define the effect of diet on lung function.

	Childhood		Adulthood	
	Effect	Evidence	Effect	Evidence
New born Breast feeding	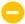 [185,186,187]	Low	?	?
Mediterranean diet	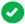 [43,188]	Very low	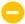 [43,170,171,188]	Strong
Fruit	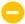 [43]	Very low	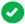 [172,173,174]	Strong
Vegetables	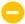 [43]	Very low	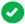 [174]	Strong
Fast food	?	?	?	?
“Western” diet	?	?	?	?
Meat	?	?	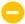 [71]	Low
Fish	?	?	?	?
Vitamin A	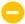 [118]	Low	?	?
Vitamin B	?		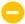 [175]	Very low
Vitamin C	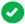 [166,189]	Strong	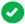 [197]	Strong
Vitamin D	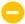 [190,191,192]	Very strong	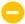 [190,191,198]	Very strong
Vitamin E	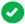 [193]	Strong	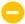 [197]	Strong
LC *n*-3 PUFA (Fish oil)	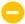 [194]	Strong	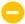 [194]	Strong
